# Supplementary material for: One-Year Follow-Up of COVID-19 Patients Indicates Substantial Assay-Dependent Differences in the Kinetics of SARS-CoV-2 Antibodies
Source: Microbiol Spectr. 2022 Oct 12;10(6):e00597-22. doi: 10.1128/spectrum.00597-22 (PMC9784763; doi:10.1128/spectrum.00597-22)
Supplement: Supplemental file 1 — Supplemental Text, Tables S1 to S9, and Fig. S1 to S4. Download spectrum.00597-22-s0001.pdf, PDF file, 1.6 MB [file spectrum.00597-22-s0001.pdf]

Supporting Information to

One year follow-up of COVID-19 patients indicates substantial assay-dependent differences in the kinetics of SARS-CoV-2 antibodies

Table of content

Table S1: Figure of merits of the SARS-CoV-2 antibody assays investigated p.2

Morbidity assessment of the Study Patients p.3

Statistics and visualization p.3

Live virus neutralisation assay p.4

Table S2: Overview of neutralisation assay parameters for interlaboratory comparison

Table S3: Neutralising antibody titres of the WHO reference panel and international standard

Patient cohort and study time points p.6

Figure S1: Overview on missed study visits out of the 145 study participants

Qualitative agreement between the assays – root mean squared differences p.7

Table S4: Overview of the qualitative agreement between the investigated serologic assays

Table S5: Root mean squared differences (RMSD) of test results for all data and visit specific

Quantitative agreement between the assays

Table S6: Performance of the assays using the first WHO reference material p.9

Figure S2: Correlogram across all time points using Spearman rank correlation p.9

Figure S3: Modified Bland-Altman plots of the Technoclone_N assay with the S-protein tests p.10

Table S7: Time dependence of median VNT, antibody levels, and median relative amount p.11

Figure S4: VNT and antibody levels over time in subgroups age, gender and disease severity, and multimorbidity p.12

Table S8. Patient's distribution regarding disease severity and morbidity p.13

Table S9: Parameters determined to represent the antibody kinetics over time p.13

Table S1: Figure of merits for the SARS-CoV-2 antibody assays investigated based on the package inserts.

	Quantitative assays						Qualitative assays	
	**Diasorin** SARS-CoV-2 TrimericS IgG	**Siemens** SARS-CoV-2 IgG (sCOVG)	**Abbott** SARS-CoV-2 IgG II Quant.	**Roche** Elecsys Anti-SARS-CoV-2 S	**Techno-clone** Technozym RBD IgG	**Techno-clone** Technozym NP IgG	**Abbott** SARS-CoV-2 IgG	**Roche** Elecsys Anti-SARS-CoV-2
Abbreviated	Dia_S	Sie_S	Abb_S	Roc_S	Tec_S	Tec_N	Abb_N	Roc_N
Detected antibodies (class)	IgG	IgG	IgG	high affinity, including IgG	IgG	IgG	IgG	antibodies including IgG
Detected epitope	S-trimer	RBD of S1	RBD of S1	RBD of S1	RBD of S1	N protein	N protein	N protein
ULOQ (BAU/ml)	2080	3240	5680	257	600	400	n.a.	n.a.
extended ULoQ (BAU/ml)	Not available	16200 (1:5 predil.)	11360 (1:2 predil.)	25720 (1:100 predil.)	6000 (1:10 predil.)	4000 (1:10 predil.)	n.a.	n.a.
Negative	<33.8 BAU/ml	<21.6 BAU/ml	<7.1 BAU/ml	<0,8 BAU/ml	<=30 BAU/ml	<=20 BAU/ml	<1.4 S/C	<1.0 S/C
Positive	>=33.8 BAU/ml	>=21.6 BAU/ml	>=7.1 BAU/ml	>=0,8 BAU/ml*	>30 BAU/ml	>20 BAU/ml	≥ 1.4 S/C	≥1.0 S/C
Traceability to WHO Standard	Yes; BAU/ml= AU/ml*2.6	Yes; BAU/ml= 21.6*U/ml	Yes; BAU/ml= AU/ml*0.142	Yes; BAU/ml= 1.0288 U/Ml	Yes; BAU/ml= U/ml*6	Yes; BAU/ml= U/ml*4	n.a.	n.a.
Neutralizing antibodies	yes microneutralization test	yes, VNT	yes, PRNT	yes, VSV pseudo-neutrali-zation test	no	No	No	Yes, VSV
Sensitivity (>14/15d)	98.7 (n=155)	14-20 d: 91.1 (n=79)	99.37 (n=158)	98.8 (n=1423)	100 (n=18)	100 (n=18)	100 (n=88)	99.5 (n=185)
Specificity	99.5 (n=1899)	99.9 (n=1995)	99.55 (n=2008)	99.98 (n=5991)	99.3 (n=456)	99.8 (n=456)	99.63 (n=1070)	99.8 (n=10453)
Our instrument	LIAISON XL	Centaur XP	Architect	Cobas e602	BEP 2000	BEP 2000	Architect	Cobas e602
Package insert version	2020-12	2020-10	2021-06	2021-05	06-04-2021, Rev.3	06-04-2021, Rev.3	April 2020	2021-03

* Roche states at 0.8 BAU/ml a positive predictive value (PPV) for the presence of neutralizing antibodies of 96.27% and at 15 BAU/ml a PPV of 99.1%.

Morbidity assessment of the study patients

The following areas of morbidity were evaluated for each patient: infectious disease, malignancy, blood and blood forming organs / immune system, endocrine / nutritional / metabolic disorder, behavioural morbidity, nervous system, eyes and adnexa, circulatory system, respiratory system, digestive system, skin and subcutaneous tissue, musculoskeletal and connective tissue, genitourinary system, renal system, and immunodeficiency. Patients bearing ≥ 4 morbidities were considered as polymorbid.

Statistics and visualization

Modelling the 12 months VNT. In the first step of developing a prediction model for the neutralization titre at the last visit, a random intercept and slope model was used to estimate an intercept (rescaled to day 100) and a slope for each patient, while borrowing strength across patients and taking irregular spacing of measurements and missing measurements (assuming missingness at random) into account. For this model, neutralization titres and assay test results were log-transformed. Conditional residuals were checked for symmetry and influential observations. Medians and quartiles of back-transformed intercept and slope (referring to 30 days) across patients are reported.

The estimated assay intercepts at day 100 and slopes were then used as independent variables in linear regression models with the log of neutralization titre at the last visit as dependent variable. Due to zero values for neutralization titre, a shift of 4 was added before log-transformation such that residual distributions for all independent variables were approximately normal. The deletion of one potentially influential observation (ID 4) did not change the results to a relevant degree (results not reported).

Visualization. Bland-Altmann plots were generated with ggplot2 package in R version 4.0.3. Due to skewed distributions, the ratio of the two tests evaluated was plotted against their geometric mean. Prior to the analysis, a ratio between 70% and 130% was considered acceptable. The missing plot (Figure S1) was generated using the missingplot package (https://cran.r-project.org/web/packages/VIM/citation.html) and was arranged afterward by GIMP (Version 2.10.30). Correlogram analysis (Figure S2) and visualization is based on the corrgram package (https://cran.r-project.org/web/packages/corrgram/index.html).

Live virus neutralisation assay

All relevant assay parameters are listed in Table S2. To ensure specificity of the VNT assay we have tested 100 human serum samples which were collected before October 2019. The serum samples were obtained from our internal screenings for neutralising antibodies against rabies in vaccinated personnel. None of these tested serum samples showed any neutralising activity at the lowest serum dilution (1 in 4) against SARS-CoV-2. Based on these initial tests we have set the cut-off at 4 since we have used the assay in the beginning of the pandemic primarily for seroprevalence studies to ensure sufficient sensitivity.

Table S2. Overview of neutralisation assay parameters for interlaboratory comparison.

Cell line	Cell number per well	Virus per well	Incubation time	Assay read out	Virus lineage	Cut-off
Vero E6	*25 000*	*100 $TCID_{50}$*	4 days at 37°C, 5%CO_2	CPE	Clade B.1*	<1:4

$TCID_{50}$ Tissue culture infectious dose; CPE Cytopathic effect, * Isolate GISAID accession ID EPI_ISL_583577

To validate the NT assay we have obtained the WHO reference panel and reference standard for anti SARS-CoV-2 antibodies (NIBSC, UK; WHO reference Number: WHO/BS/2020.2402). The WHO assigned an arbitrary unitage to the reference standards to establish international units for neutralizing antibodies. The VNTs obtained by us are shown in comparison to the international units (IU) in Table S3. Each standard was set up in triplicate, and the experiment was repeated three times by two different operators. The results correlated well with the assigned IU, the low positive sample (20/140) was well above the cut-off and the negative sample showed no neutralising activity at the lowest serum dilution. Since neutralising antibody testing is not standardised those reference materials should help harmonising results to reduce inter-laboratory variation. However differences between laboratories are inherent (i.e. virus isolate, cell lines, procedure, readout, result reporting) since no standardised method has been described.

Table S3. Neutralising antibody titres of the WHO reference panel and international standard.

	**International standard**	**WHO reference panel**				
NIBSC code	*20/136*	*High (20/150)*	Mid (20/148)	Low S, high N[#] (20/144)	Low (20/140)	Negative (20/142)
Neutralising antibody (IU/ml)	1000	1473	210	95	44	negative
**Mean Neutralising antibody titre**	**320**	**n.a.[*]**	**64**	**32**	**16**	**<4**

[#] S (Spike), N (Nucleoprotein)

[*] we observed cytotoxic effects with this sample (pooled plasma), we were unable to assign a titre/value

Patient cohort and study time points

A total number of 145 patients participated in the study. Complete data set (*i.e.* participation at all 4 visits over a year) is available from 83 persons. Data from all but one visit is available from 41 individuals (15 were included later than V0, two and four missed V1 and V2, respectively, and 20 did not show up the voluntary visit after a year). In summary, 122, 138, 125, and 107 patients showed up for V0, V1, V2 and V3, respectively. 36 participants had received a vaccination against SARS-CoV-2 between V2 and V3. However, 5 of them were still included in our study and considered as unvaccinated as their first vaccination was ≤3 days prior to the study-visit. Thus, we were able to study antibody levels of 76 individuals over one year: 59 with data obtained at all visits and 15 only missing one visit, Figure S1). Complete data set on all antibody assays investigated is available from 453 out of 461 study samples and VNT data from 460 out of 461 (cf. Table S4). Missing data was considered non-informative in our model calculations, see statistics for details.

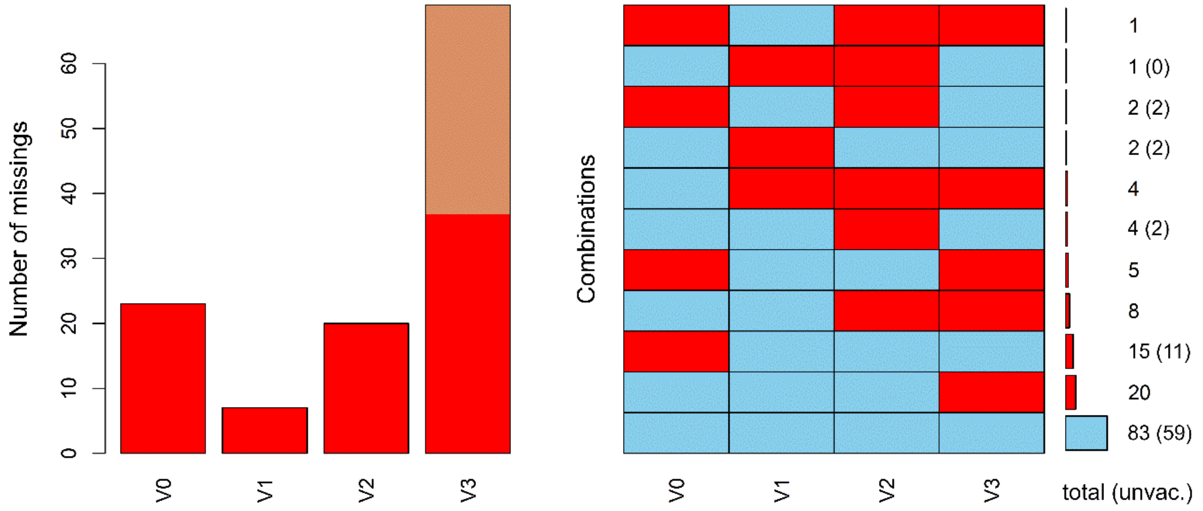

Figure S1. Overview on missed study visits out of the 145 study participants. Left: Number of missings for each visit. The brown bar at V3 indicates participants that showed up at V3 but were excluded due to vaccination prior to the study visit (n=31). Right: distribution on participants regarding showing up at a visit (blue) or missing (red). The numbers in brackets represent the unvaccinated participants.

Qualitative agreement between the assays – root mean squared differences

In order to quantify the agreement of each assay with an average across assays the following approach was used. First, for each sample the arithmetic mean across all non-missing assay test results (0 or 1 for negative or positive, Table S4) was calculated (assuming non-informative missingness) resulting in a proportion positive between 0 and 1. Next, for each assay, the root mean square difference (RMSD) of the test results and this mean was calculated as the square root of the mean (across all samples with at least 4 non-missing assay results) of squared differences between the observed result (0 or 1) and the mean assays result. These calculations were performed using SAS 9.4 (SAS Institute Inc., 2016).

Considering all samples investigated, the assays of Roche_N, Roche_S, Abbott_S, Siemens_S and Diasorin_S exhibit similar root mean squared difference (RMSD) ranging from 0.15 to 0.17, while the Technoclone_N, Technoclone_S, and Abbott_N gave more often deviant results (RMSD 0.22-0.33) from the calculated mean of all assays. Assays generally performed more comparable at the V0 visit (RMSD 0.06-0.18 for all assays) and the least one year after disease onset (RMSD 0.22-0.56), indicating that independent from the used assay, a qualitatively identical result is likely at V0, but not at V3 (Table S5). In accordance with the lowest positivity rates at V3 for the Technoclone_N and Abbott_N assay, the RMSD is 0.514, and 0.560, respectively.

Table S4. Overview of the qualitative agreement between the investigated serologic assays. Positive results, indicating the presence of antibodies against SARS-CoV-2, are given as “1”, while the negative ones are given as “0”. Frequency count represents the number of samples showing the respective constellation. All samples (n=461) at all visits points are summarized.

Spike Protein specific assays					Nucleocapsid specific assays						
Abb_S	Dia_S	Roc_S	Sie_S	Tec_S	Abb_N	Roc_N	Tec_N	Frequency Count	Percent of Total Frequency	number non-missing[a]	proportion positive[b]
1	1	1	1	1	1	1	1	322	69.85	8	1.000
1	1	1	1	1	0	1	0	41	8.89	8	0.750
0	0	0	0	0	0	0	0	17	3.69	8	0.000
1	1	1	1	1	1	1	0	15	3.25	8	0.875
1	1	1	1	0	0	1	0	13	2.82	8	0.625
1	1	1	1	1	0	1	1	9	1.95	8	0.875
1	1	1	1	0	1	1	0	7	1.52	8	0.750
1	1	1	0	0	0	1	0	4	0.87	8	0.500
1	1	1	1	0	1	1	1	3	0.65	8	0.875
0	0	1	0	0	0	0	0	3	0.65	8	0.125
1	1	1	0	0	1	1	1	2	0.43	8	0.750
1	1	1	0	0	1	1	0	2	0.43	8	0.625
1	0	1	1	0	0	1	1	2	0.43	8	0.625
1	0	1	1	0	0	1	0	2	0.43	8	0.500
1	0	1	0	0	0	1	1	2	0.43	8	0.500
0	0	0	0	0	1	0	1	2	0.43	8	0.250
1	1	1	1	0	0	1	1	1	0.22	8	0.750
1	0	1	1	1	0	1	0	1	0.22	8	0.625
1	0	1	0	0	1	1	1	1	0.22	8	0.625
1	0	1	0	0	0	1	0	1	0.22	8	0.375
0	0	1	0	0	0	1	1	1	0.22	8	0.375
0	0	1	0	0	0	1	0	1	0.22	8	0.250
0	0	0	0	0	0	0	1	1	0.22	8	0.125
1	1	1	#N/A	1	1	1	1	1	0.22	7	1.000
1	1	1	#N/A	0	0	1	1	1	0.22	7	0.714
1	#N/A	1	1	1	1	1	1	1	0.22	7	1.000
1	1	1	1	#N/A	0	1	#N/A	2	0.43	6	0.833
1	1	1	0	#N/A	0	1	#N/A	1	0.22	6	0.667
1	0	1	0	#N/A	0	0	#N/A	1	0.22	6	0.333
1	1	1	#N/A	#N/A	#N/A	#N/A	#N/A	1	0.22	3	1.000

[a] number of non-missing results among eight qualitative test results

[b] proportion of positive among non-missing test results

#N/A not available due to insufficient sample volume

Table S5. Root mean squared differences (RMSD) of test results for all data and visit specific. The closer the RMSD is to 0, the better the results of the respective test agree with the other tests (see also Table S4), the closer to 1, the more the results of the respective test deviates. Corresponding positivity rates are calculated from all samples available at the respective time point and assay.

Assay	all	V0		V1		V2		V3	
	RMSD	RMSD	Pos. rate, %	RMSD	Pos. rate, %	RMSD	Pos. rate, %	RMSD	Pos. rate, %
Abb_S	0.145	0.059	93	0.107	94	0.172	95	0.231	96
Dia_S	0.149	0.074	93	0.123	93	0.166	93	0.230	91
Roc_S	0.166	0.098	94	0.130	95	0.194	97	0.245	97
Sie_S	0.156	0.059	93	0.121	92	0.200	89	0.222	92
Tec_S	0.215	0.147	90	0.191	87	0.230	82	0.305	80
Abb_N	0.309	0.117	93	0.191	89	0.329	74	0.560	38
Roc_N	0.149	0.059	93	0.107	94	0.178	96	0.236	96
Tec_N	0.331	0.184	92	0.294	82	0.346	73	0.514	47

Quantitative agreement between the assays

Table S6: Performance of the assays using the first WHO reference material. The reference material (1000 BAU/ml) was serially diluted with negatively tested serum to the expected concentrations. Results of these samples measured with the respective assays are given in BAU/ml. Abb_S (Abbott SARS-CoV-2 IgG II Quant.), Dia_S (Diasorin SARS-CoV-2 TrimericS IgG), Roc_S (Roche Elecsys Anti-SARS-CoV-2 S), Sie_S (Siemens SARS-CoV-2 IgG), Tec_S (Technozym RBD IgG), Tec_N (Technozym NP IgG).

expected	Abb_S	Dia_S	Roc_S	Sie_S	Tec_S	Tec_N
1000	947	1000	877	724	>600	>400
310	241	325	233	#N/A	299	195
100	83	135	85	#N/A	99	73
31	25	35	25	20	28	22
10	10	15	9	8.7	12	8.5
3.1	3.4	7.1	2.7	2.8	6.4	4.6

#N/A not available due to insufficient sample volume

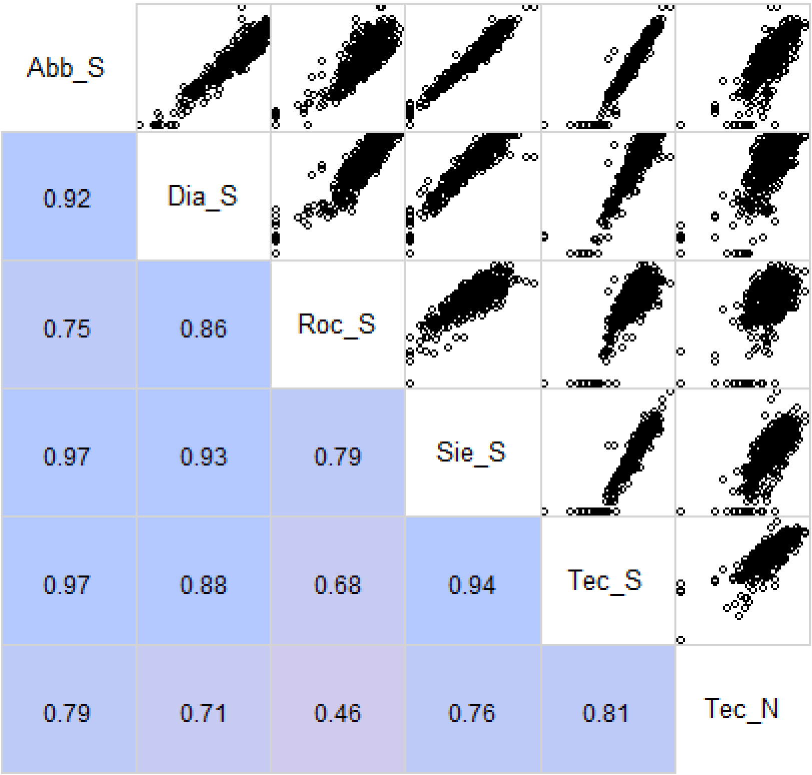

Figure S2. Correlogram across all time points using Spearman rank correlation. Scatter plots are on log-scales. Tec_N (Technozym NP IgG), Tec_S (Technozym RBD IgG), Abb_S (Abbott SARS-CoV-2 IgG II Quant.), Roc_S (Roche Elecsys Anti-SARS-CoV-2 S), Sie_S (Siemens SARS-CoV-2 IgG), Dia_S (Diasorin SARS-CoV-2 TrimericS IgG).

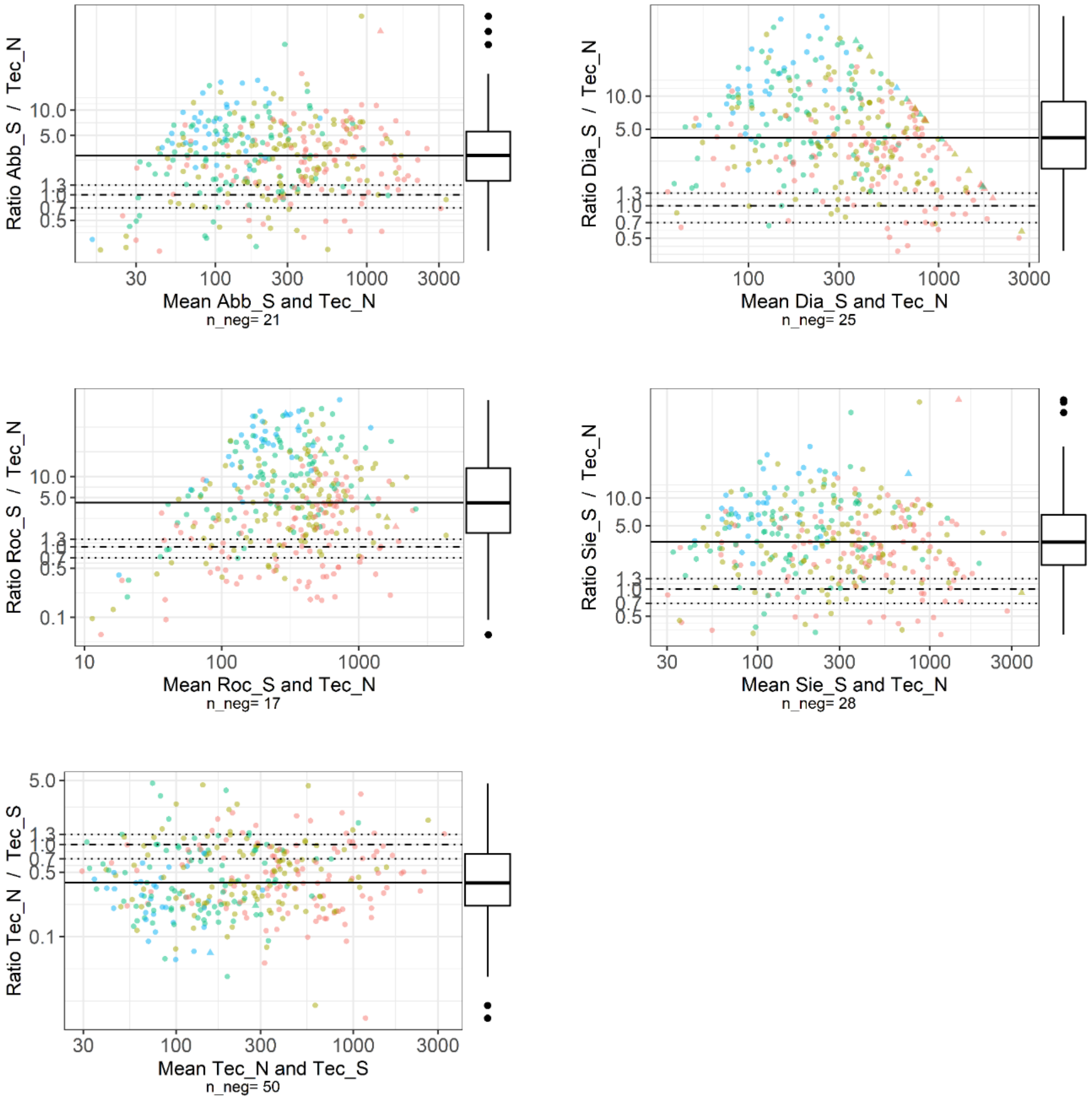

Figure S3. Modified Bland-Altman plots of the Technoclone_N assay with the S-protein tests. x-axis (logarithmized): geometric mean of both methods (in BAU/ml), y-axis (logarithmized): ratio of the two methods, continuous line= median ratio; dashed line at 1.0 = equivalence between both methods, dotted lines= predefined boundaries of acceptance (± 30% of equivalence), colour codes: red=V0, lime=V1, green=V2, blue=V3, ▲= observation above upper limit of quantification for at least one of the two measurements, n_negative= observations not displayed due to negative result in at least one assay.

Table S7. Time dependence of median VNT, median antibody levels, and median relative amount.

	V0§	V1	V2	V3#
VNT, titre	128	64	48	32
(Q1; Q3)	(48; 256)	(24; 128)	(16; 96)	(16; 64)
relative amount, %*		50%	50%	25%
(Q1; Q3)	-	(50;100)	(25;50)	(25;50)
Abbott_S, BAU/ml	671	326	175	129
(Q1; Q3)	(194; 1672)	(88; 733)	(63; 367)	(56; 267)
relative amount, %*		57%	27%	20%
(Q1; Q3)	-	(47;68)	(21;40)	(12;31)
Diasorin_S, BAU/ml	772	602	380	273
(Q1; Q3)	(273; 1412)	(186; 1150)	(156; 762)	(126; 605)
relative amount, %*		82%	51%	36%
(Q1; Q3)	-	(69;96)	(38;75)	(27;51)
Roche_S (BAU/ml)	421	501	653	633
(Q1; Q3)	(102; 1185)	(159; 1340)	(163; 1535)	(206; 1431)
relative amount, %*		117%	120%	125%
(Q1; Q3)	-	(97;148)	(100;195)	(80;191)
Siemens_S (BAU/ml)	685	418	235	173
(Q1; Q3)	(193; 1328)	(105; 880)	(76; 516)	(65; 367)
relative amount ,%*		65%	34%	23%
(Q1; Q3)	-	(53;82)	(27; 48)	(16;33)
Technoclone_S (BAU/ml)	584	302	159	82
(Q1; Q3)	(165; 1347)	(69; 567)	(56; 327)	(38; 158)
relative amount, %*		55%	28%	14%
(Q1; Q3)	-	(44;64)	(20;38)	(10;25)
Technoclone_N (BAU/ml)	272	131	53	16
(Q1; Q3)	(102; 483)	(38; 286)	(19; 115)	(8; 36)
relative amount, %*		48%	18%	7%
(Q1; Q3)	-	(38;58)	(13;27)	(5;11)

* Relative amount is calculated as the median from all the patient specific relative kinetics: Antibody levels at V1, V2, and V3 were referenced to the patient's V0 level prior to calculating the visit specific median and quartiles.

§ Patients with negative antibody response at V0 were excluded from analysis for all further time points for the respective assay.

# Patients having already received a vaccination against SARS-CoV-2 at V3 were excluded at the last time point.

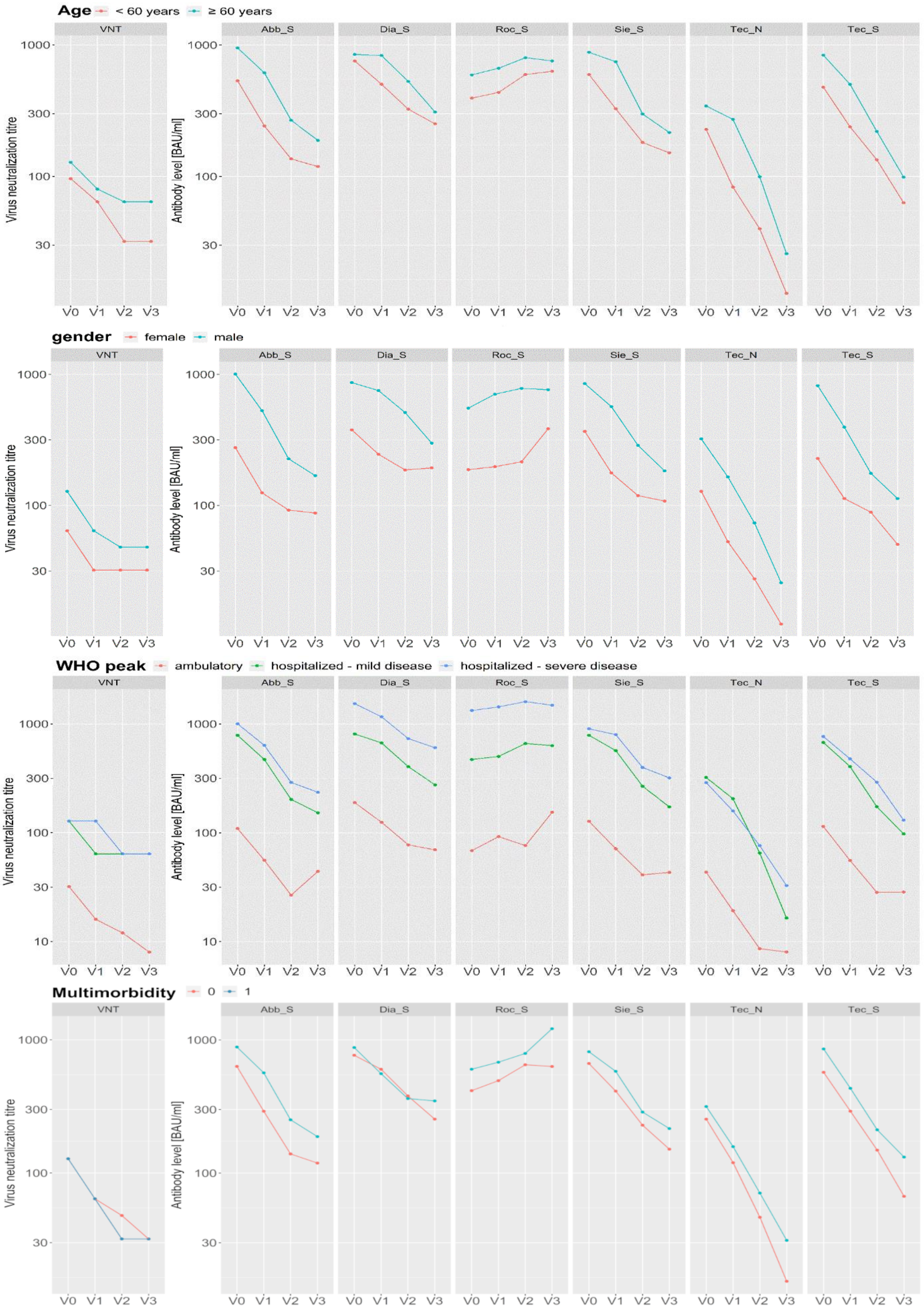

Figure S4. VNT and antibody levels over time in the subgroups age, gender, disease severity, and multimorbidity. Time dependence of median antibody levels 60 days (V0), 3 months (V1), 6 months (V2), and 12 months (V3) after disease onset is shown as median (titre steps or BAU/ml) of all samples available at the specific time point. Patients without formation of antibodies at V0 were excluded throughout this analysis, and patients having received vaccination against SARS-CoV-2 prior to V3 were not considered for analysis at the 12 months visit only.

Table S8. Patient's distribution regarding disease severity and morbidity. Absolute numbers (n) and corresponding relative amount (%) are given for each category.

Multimorbidity	Ambulatory		Hospitalized (mild)		Hospitalized (severe)	
	n	%	n	%	n	%
No	34	29	63	53	22	18
Yes	2	8	13	50	11	42

Table S9. Parameters determined to represent the antibody kinetics over time. Calculation is based on the measured antibody levels at V0, V1 and V2 across 76 patients.

Assay	Intercept at day 100*			Slope for 30 days#		
	Median	Lower Quartile	Upper Quartile	Median	Lower Quartile	Upper Quartile
VNT	87.8	28.8	139.7	0.855	0.822	0.884
Abb_S	452.4	135.9	957.4	0.759	0.721	0.814
Dia_S	642.8	213.0	1323.3	0.871	0.838	0.924
Roc_S	553.5	159.9	1239.5	1.064	1.035	1.108
Sie_S	531.0	154.9	1171.5	0.802	0.776	0.835
Tec_N	139.6	56.6	309.8	0.696	0.654	0.743
Tec_S	391.3	122.2	750.4	0.775	0.740	0.817

* Antibody level (given as neutralization titre or serological antibody levels in BAU/ml) 100 days after disease onset

Estimated relative change in antibody level per 30 days
